# ‘Leaves and Eats Shoots’: Direct Terrestrial Feeding Can Supplement Invasive Red Swamp Crayfish in Times of Need

**DOI:** 10.1371/journal.pone.0042575

**Published:** 2012-08-03

**Authors:** Jonathan Grey, Michelle C. Jackson

**Affiliations:** 1 Department of Physiological Ecology, Max Planck Institute for Limnology, Plön, Schleswig-Holstein, Germany; 2 School of Biological and Chemical Sciences, Queen Mary University of London, London, England; University of California, Berkeley, United States of America

## Abstract

We used stable isotope analyses to characterise the feeding dynamics of a population of red swamp crayfish in Lake Naivasha, Kenya, after the crash of submerged macrophytes and associated macroinvertebrates, and during a natural draw-down of the lake water level. We expected a heavy reliance upon a diet of detrital matter to sustain the population as a consequence, and indeed, for the majority of the crayfish population caught from the lake, we saw a concomitant shift in isotopic values reflecting a dietary change. However, we also caught individual crayfish that had occupied the footprints of hippopotamus and effectively extended their range beyond the lake up to 40 m into the riparian zone. Isotopic analysis confirmed limited nocturnal observations that these individuals were consuming living terrestrial plants in the vicinity of the footprints. These are the first empirical data to demonstrate *direct* use of terrestrial resources by an aquatic crayfish species and further highlight the traits that make red swamp crayfish such opportunistic and successful invaders.

## Introduction

Crayfishes form a globally diverse group of invertebrates comprising some 600+ species [1] which vary considerably in their ecological and life history traits [2], [3]. Within the group are those species which can be considered as highly imperilled and at the other end of the spectrum are those considered as pernicious invasive species which have negatively affected freshwater ecosystems throughout the world [4], [5]. Amongst the latter, the red swamp crayfish (*Procambarus clarkii*) is the most widely distributed crayfish and considered a globally important invader of freshwater ecosystems because of its ecological plasticity [6], [7]. It tends to be the largest invertebrate wherever it is introduced, causing cascading effects and impacting upon ecosystem structure and processes [8]. Commonly reported impacts include a reduction or extirpation of native macrophytes [9], alteration of community assemblages, both invertebrate and vertebrate [10], [11], playing host and thus vector of the water mold, *Aphanomyces astaci*, otherwise known as crayfish plague [12], and destruction of banks via burrowing [10]. Thus, after successful establishment and by invoking considerable ecological change, *P. clarkii,* is often considered as an ecosystem engineer and keystone species [13], [14].

From the 1960 s to present, red swamp crayfish have been introduced to multiple locations throughout East Africa to enhance fisheries and in an attempt to control schistosomiasis because they feed on snail vectors that carry schistosome larvae [15], [16]. In 1970, *P. clarkii* was introduced to Lake Naivasha, Kenya, where it underwent cyclic population fluctuations in relation to the abundance of submerged macrophytes; there was an inverse correlation between *P. clarkii* abundance in the lake (sampled as importance value in the diet of large-mouthed bass, *Micropterus salmoides*) and the area covered by submerged plant beds [17], [18]. Smart et al. [19] used feeding experiments to demonstrate that *P. clarkii* preferentially grazed upon native species of submerged macrophyte in Lake Naivasha, especially *Potamogeton octandrus*, and only preyed upon invertebrates and carrion opportunistically. The hydrological regime of Lake Naivasha is quite unstable and the gradient of the littoral benthos is very shallow [20]; thus, any small changes in water level exacerbate the stress on the littoral where the majority of macrophytes should be found, compounding problems for the crayfish finding food. Since submerged macrophytes are the principle habitat for alternative invertebrate prey, there is little left for crayfish to feed on when the macrophytes are reduced except for plant detritus in the sediments [19]. However, populations of *P. clarkii* typically rebounded rapidly with resurgence of macrophytes in Lake Naivasha, up until they came into direct conflict with another introduced species, the common carp (*Cyprinus carpio*): benthic, omnivorous carp may compete directly with red swamp crayfish for invertebrate prey and prevent re-establishment of macrophytes both directly via benthic disturbance and indirectly via maintenance of turbidity in the water column [13], [21], [22]. This ability to rapidly re-establish a dense population led us to examine the diet of the crayfish by stable isotope analysis [23] during a period when macrophytes declined (to become effectively absent) and prior to carp establishment to determine whether those detrital resources were responsible for stabilising the population (*sensu* Moore et al. [24]).

## Materials and Methods

### Ethics Statement

All animal work was conducted in accordance to national and international guidelines to minimise discomfort to animals (Schedule 1 of the Animals [scientific procedures] Act, 1986). Since there were no regulated procedures involved, the Max Planck Institute for Limnology board reviewing the project declared there was no requirement for ethics approval.

The necessary permits were obtained for the described field studies from the National Council for Science and Technology, Kenya: NCST 5/002/R/020-D (formerly OP/13/001/12C46). We collected samples from Lake Naivasha along the relatively undisturbed eastern shoreline below the Kenya Wildlife Services training camp (0′44′42S, 36′25′21E) in three consecutive years (2001-03) during a two week period each July; firstly when submerged macrophytes were present, secondly when they were absent and the littoral zone was drawn down ∼100 m horizontal distance, and thirdly when the water level had recovered but macrophytes were still absent. The receding water level left exposed sediments which were initially sparsely colonised by *Ludwigia* spp. and *Polygonum* spp., eventually to be followed by the Kikuyu grass *Pennisetum clandestenum*. Representative samples of dominant plants, detrital material and fine sediments were taken manually from 10 areas along the lake shore. Since the lake is typically turbid (Secchi depth <25 cm; pers. obs.) and the fine benthic sediments prone to resuspension, periphytic primary production is negligible. We have shown previously that because of the substantial hippopotamus (hereafter hippo) population size, their dung is a considerable allochthonous resource to the lake (Hippo Mediated Organic Matter - HMOM), being composed primarily of partially ruminated, short-sward C4 grasses such as *P. clandestenum* [25]. HMOM was collected onshore at lake entry and exit points which were clearly visible from the density of hippo tracks and propensity to mark their passing by dung scattering. Oligochaetes and chironomids were the only potential invertebrate prey to be found in sufficient abundance to be sampled for stable isotope analysis; they were removed from sediments after sieving with a 500 µm sieve and a minimum of 20 individuals pooled to form each replicate (n = 5). Crayfish (standardised for carapace length 45–60 mm and no exterior damage i.e. missing limbs etc) were collected using baited traps or net sweeps.

A fortuitous finding when the lake was naturally drawn down (i.e. only in the middle year) led us to manually search for crayfish burrows in hippo footprints. Because the gradient of the littoral/riparian was so shallow, hippo footprint depressions (up to 50 cm deep) were above the waterline (to a distance of 40 m) but below the water table. Approximately 30% of these highly turbid, miniature pools contained crayfish occupying shallow horizontal burrows. Observations after dark revealed these crayfish were clambering out from the footprints and apparently grazing upon the terrestrial pioneer plant species that had rapidly colonised the former lake bed. Consequently, 16 individual crayfish were also collected from this temporary habitat. All samples were prepared for stable carbon and nitrogen isotope analyses (following Britton et al. [26]). We present the data via standard isotope bi-plots and calculate relative contributions to crayfish biomass from the putative basal resources using mixing models (Stable Isotope Analysis in R - SIAR: [27]) and appropriate fractionation factors with measures of variability derived for Crustacea from the literature [28]–[30]. These were: 1.93±0.82‰ and 2.04±0.11‰ for δ^13^C, and 3.88±2.23‰ and 4.24±0.99‰ for δ^15^N for crustaceans fed upon animal and plant matter, respectively. Bayesian models such as SIAR have greater statistical power, allowing users to incorporate such variation in discrimination factors. In addition, we used our stable isotope data to calculate population level metrics bootstrapped for the minimum population size (16) in SIBER (Stable Isotope Bayesian Ellipses in R), and a sample size corrected version of the standard ellipse area (SEA_c_) to circumvent the bias that arises when sample sizes are small [31]; the latter can be equated to a measure of the crayfish mean core ‘isotopic niche’ [22], [31].

## Results and Discussion

**Figure 1:**
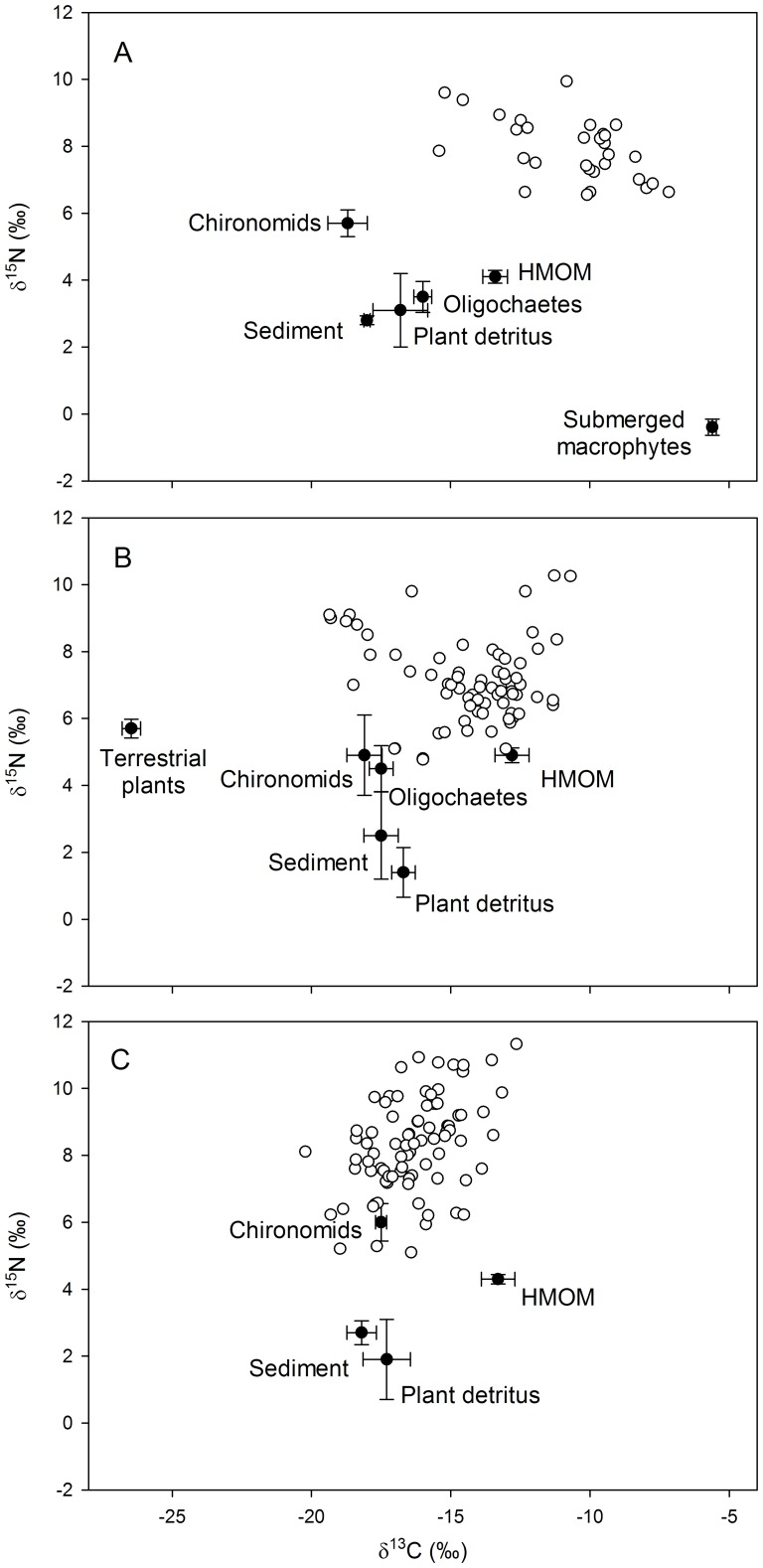
Stable isotope bi-plots of the Lake Naivasha food web derived from samples collected during July in A) 2001, B) 2002, and C) 2003. Open symbols represent individual *Procambarus clarkii* relative to the putative basal resources (solid symbols: means ±SE from n = 5 to 15) which were present and sufficiently abundant during collection to be analysed for stable isotopes. HMOM – Hippo Mediated Organic Matter.

If individual red swamp crayfish assimilated everything in equal proportions, then their isotopic values should tend to be similar and, therefore, the population should exhibit a narrow isotopic niche. Throughout the period of study, the basal resource isotope values in Lake Naivasha remained relatively constant [22], yet individual crayfish exhibited considerable intraspecific isotopic variability (Fig. 1a–c; Table 1 metrics) indicating a broad choice in diet but with some individuals apparently specialising upon different resources [32], and supporting the findings of previous dietary studies from gut content analyses [19], [28]. Vegetable matter in various forms dominated the diet. When submerged macrophytes were present in 2001 only, they contributed ∼17% (range 7–27%) to crayfish biomass, with ∑61% comprising ‘processed’ plant matter from HMOM and mixed detritus in the sediments (Table 1). The lack of animal prey in the diet likely contributed to the relatively narrow NR (2.95‰) and thus a modest niche value (5.56‰^2^). The greatest isotopic niche area (SEA_c_  = 8.20‰^2^) was recorded in the intermediate year when the water level was lowest, the macrophytes had disappeared, and we found individual crayfish inhabiting the hippo footprints. This was despite the contributory proportions estimated from SIAR being rather similar to the previous year (with a shift in emphasis from HMOM to detritus) if the population was considered as a whole (Table 1).

**Table 1:** Mean percentage contributions to *Procambarus clarkii* biomass derived from SIAR (low and high high-density regions) for six putative resources when available in the different years (N/A – not available), and for 2002 calculated separately for the sub-populations found onshore and offshore.

Year	Sub-pop^n^	Resources used in SIAR modelling of *P. clarkii* biomass						Mean population metrics		
		Submergedmacrophytes	Terrestrialplants	Hippo Dung(HMOM)	Mixeddetritus	Chironomids	Oligochaetes	CR_b_(‰)	NR_b_(‰)	SEA_c_(‰^2^)
2001	Lake	17.0 (7.2–27.4)	2.6 (0.0–7.3)	55.8 (28.9–80.5)	5.4 (0.0–15.3)	12.3 (0.0–29.8)	6.9 (0.0–19.0)	7.08	2.95	5.56
2002	Lake	N/A	3.0 (0.0–7.6)	21.3 (10.7–31.6)	54.1 (44.5–63.7)	9.3 (0.0–22.2)	12.2 (0.0–28.1)	6.99	4.36	8.20
	Offshore	N/A	1.0 (0.0–2.6)	33.9 (27.7–40.0)	58.3 (50.5–66.0)	3.0 (0.0–8.0)	3.7 (0.0–9.6)	3.99	3.95	3.55
	Onshore	N/A	26.6 (18.0–36.3)	8.4 (0.0–20.3)	29.1 (10.4–48.2)	17.9 (0.0–37.1)	18.1 (0.0–36.9)	3.70	4.56	5.91
2003	Lake	N/A	20.4 (9.6–30.3)	23.1 (0.6–42.0)	30.5 (18.2–42.6)	26.1 (1.7–50.0)	N/A	5.12	5.61	6.68

The mean bootstrapped population metrics calculated from SIBER are: CR_b_ – carbon range; NR_b_ – nitrogen range; and SEA_c_ – corrected standard ellipse area.

**Figure 2:**
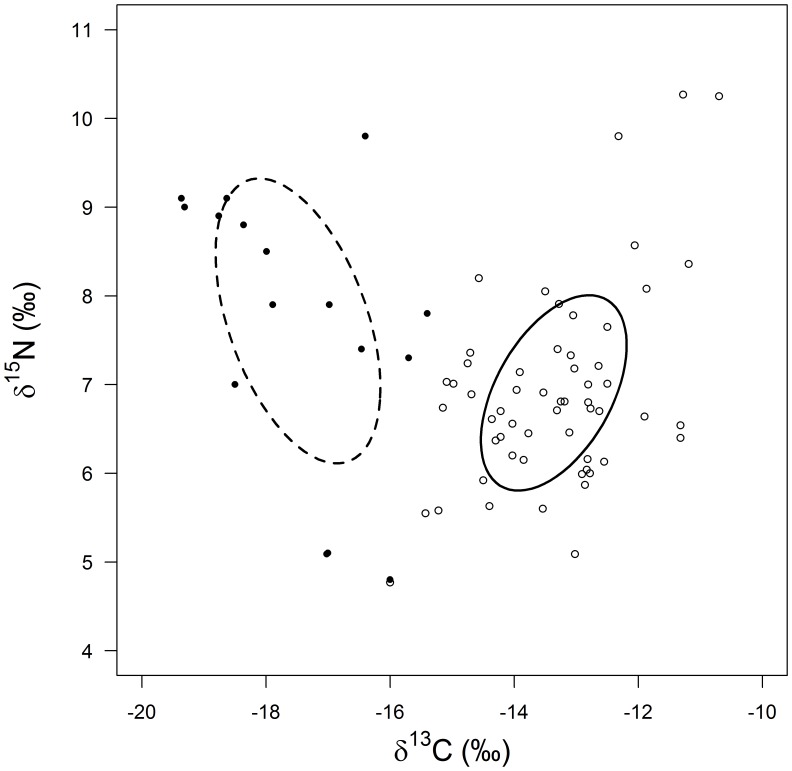
Stable isotope bi-plot of individual *Procambarus clarkii* collected at Lake Naivasha during 2002. The sub-population collected from hippo footprints onshore are represented by solid symbols, and those from within the lake by open symbols. The corrected standard ellipse area (SEA_c_) is plotted for each sub-population.

A more detailed analysis of the crayfish sub-populations found in 2002 demonstrated that there was virtually no isotopic overlap and certainly no overlap in niche space as measured by SEA_c_ between individuals caught from the footprints (onshore) and those caught in the lake (offshore; Fig. 2; Table 1). SIAR modelling revealed that terrestrial plants (∼27%) were an important component of crayfish biomass collected from the hippo footprints, almost equal to that derived from mixed detritus (∼30%), and that the isotopic niche of the onshore population was larger than that from within the lake, primarily because they had direct access to terrestrial plants (Fig. 2; Table 1). The contribution from terrestrial plants to lake-dwelling crayfish was negligible in 2002 as it had been in 2001. Those pioneer plants which had extended across the drawdown zone were effectively inundated with the recovery of lake water level by the time we sampled in 2003, and accessible to all the crayfish residing back in the lake resulting in an estimate of ∼20% biomass contribution. We cannot tell whether the crayfish directly consumed the plants or whether they assimilated the carbon indirectly as the plants became a part of the detritus pool, although we would argue that either way, that plant material should be considered an allochthonous resource. In 2003, the population niche was smaller (6.68‰^2^) than in 2002 reflecting a return toward resources more readily available within the lake (mixed detritus and HMOM ∑53.6%).

An assumption with isotope mixing models is that the consumer organism is in isotopic equilibrium with its diet, which is of course rarely met especially when considering opportunistic omnivores [23]. However, because there was no overlap in isotopic niche with crayfish from the lake, it does imply that the crayfish that we caught from the hippo footprints must have been ‘resident’ in this temporary habitat and/or assimilating terrestrial resources for some time. From diet switch feeding trials with similar organisms [33] and considering the water temperature of Lake Naivasha is typically >20°C, it is likely that the crayfish had been grazing terrestrial plants for >1 month.

Our study confirmed that plant-derived material contributed a greater proportion to the biomass of *P. clarkii* than animal prey, and that detritus apparently helped stabilise the population (*sensu* Moore et al. [24]). However, the most striking aspect of this study is that even in aquatic ecosystems where resources are heavily reduced and resource competition thus presumably very high, tenacious invaders such as red swamp crayfish may prevail by opportunistically inhabiting temporal pools and/or *directly* accessing what would be considered allochthonous resources; i.e. terrestrial vegetation, in a similar manner to hippo. The ability of *P. clarkii* to use ephemeral or intermittent waterbodies is well known and its popularity as an aquaculture species, and hence intentional spread, partly originates from ‘double-cropping’ alongside rice cultivation [34]. It can survive in such areas with seasonal fluctuations in water level by either burrowing to avoid desiccation, or actively dispersing across dry land for short distances [35]–[37]. While *P. clarkii* and other invasive crayfish such as *Pacifastacus leniusculus* have been shown to feed heavily on allochthonous resources when they are available *within* their aquatic habitat [28], [38], this is the first study as far as the authors are aware demonstrating red swamp crayfish leaving the aquatic habitat to forage on terrestrial vegetation. It is impossible to know whether the crayfish sampled from hippo footprints were isolated as the waterline receded, or whether they actively left the lake and effectively ‘island-hopped’ in the footprints in a manner similar to the semi-terrestrial crayfish, *Euastacus sulcatus* [39], [40]. Nonetheless the stable isotope data reveal that they had been assimilating terrestrial resources for a substantial period. We speculate that such traits emphasise further the classification of red swamp crayfish amongst the ‘most invasive’ species in the world with considerable potential to expand their range [1], [6]. Indeed, it leads us to revisit the suggestion of Lodge et al. [41] that management of such exotic species should be focussed on preventing introduction, because subsequent elimination is almost impossible.
